# The Impact of Obesity as a Peripheral Disruptor of Brain Inhibitory Mechanisms in Fibromyalgia: A Cross-Sectional Study

**DOI:** 10.3390/jcm13133878

**Published:** 2024-07-01

**Authors:** Walter Fabris-Moraes, Guilherme J. M. Lacerda, Kevin Pacheco-Barrios, Felipe Fregni

**Affiliations:** 1 Neuromodulation Center, Spaulding Rehabilitation Hospital, Harvard Medical School, Charlestown, MA 02129, USA; walter.afmoraes@fm.usp.br (W.F.-M.);; 2 Faculty of Medicine FMUSP, University of São Paulo, São Paulo 01246-903, SP, Brazil; 3 Instituto de MedicinaFísica e Reabilitação, Hospital das Clínicas HCFMUSP, Faculdade de Medicina, Universidade de São Paulo, São Paulo 04116-030, SP, Brazil; 4 Unidad de Investigación para la Generación y Síntesis de Evidenciasen Salud, Vicerrectorado de Investigación, Universidad San Ignacio de Loyola, Lima 150114, Peru

**Keywords:** fibromyalgia, obesity, TMS, compensatory mechanisms, inflammation

## Abstract

**Background/Objective:** Obesity, characterized by chronic inflammation, may serve as a surrogate marker for more dysfunctional peripheral inflammation, potentially exacerbating FM symptomatology. Given this premise, this study aimed to investigate the effects of obesity as an effect modifier on neural and clinical variables, specifically those indexing pain-compensatory mechanisms in FM symptoms. **Methods:** A cross-sectional study was conducted with 108 participants who underwent a standardized TMS protocol assessment to measure resting motor threshold (MT), intracortical facilitation (ICF), and intracortical inhibition (ICI). Clinical data were collected using Beck’s Depression Index (BDI), PROMIS, the Brief Pain Inventory (BPI), and conditioned pain modulation (CPM). Linear regression models were used to explore the relationship between these variables while examining Body Mass Index (BMI) as a potential effect modifier. If it was found to be a modifier, we stratified the sample into two groups with a BMI cutoff of 30 and performed another regression model within the subgroups. **Results:** BMI was identified as an effect modifier in the relationships between ICI and BDI, PROMIS fatigue, and CPM and in MT versus CPM. After stratification, non-obese fibromyalgia subjects demonstrated significant correlations between clinical symptoms and CPM and ICI activity. However, these correlations were absent in the obese group, suggesting obesity disrupts pain mechanisms and their compensatory effects. Higher MT values were associated with weaker endogenous pain control, particularly evident in the obese group. **Conclusions:** Obesity appears to be a significant effect modifier and delineates two patient groups across multiple clinical and neural assessments of fibromyalgia. Additionally, it suggests a role for obesity in exacerbating fibromyalgia symptoms and disrupting physiological pain-inhibitory mechanisms.

## 1. Introduction

Fibromyalgia (FM) is a common pain disorder characterized by chronic diffuse pain, and it is estimated to affect 2.7% of the global population [1,2,3]. It is also associated with several other physical and mental health problems from anxiety and depression to fatigue and sleep disturbances [2]. It primarily affects women, with a prevalence of 3:1 (female/male), and it has a higher prevalence among obese women [3]. Additionally, it is estimated that healthcare costs for subjects with FM are about three times higher than for the general population [4].

It is not clear yet the neural mechanisms leading to generalized pain in fibromyalgia. One theory pertains to emotional circuits being the leading driver [5]. However, this has been put to debate because of increasing evidence for the occurrence of inflammatory processes in the periphery and central nervous systems [5]. Therefore, understanding the cortical mechanisms of pain control in FM requires the use of multiple neurophysiological tools. One of them is transcranial magnetic stimulation (TMS) [6,7,8]. TMS-derived neurophysiological variables measuring intracortical inhibition offer valuable data. Intracortical inhibition, particularly in the context of FM, reflects the balance between inhibitory and excitatory neurotransmission within the cortex [9]. ICI is measured in the primary motor cortex, an area that has been identified as critical in pain control [10]. Alterations in intracortical inhibition have been implicated in various chronic pain syndromes, highlighting its potential relevance in FM pathophysiology [11].

Moreover, investigating other measurements of neural inhibition is critical. One important measurement is conditioned pain modulation (CPM). CPM measures a person’s capacity to inhibit pain, allowing for the examination of the descending pain modulation system [12]. We have shown significant alterations of CPM in FM when compared to healthy controls; additionally, we found evidence relating it to brain functional connectivity and cognitive impairment [12].

Although these studies support critical advances in the field, one important link is missing: the influence of peripheral factors on brain plasticity. Here, we investigated obesity as such a link. Obesity, characterized by chronic inflammation, may serve as a surrogate marker for more dysfunctional peripheral inflammation [13]. This persistent inflammatory state has the potential to induce neuroplastic changes in the central nervous system, thereby potentially exacerbating FM symptomatology [14]. To date, few studies have assessed the effects of obesity in moderating neural and clinical variables in FM symptoms.

Therefore, to address this gap in the literature, we have designed this cross-sectional study. We intend to test the hypothesis that obesity might be a disruptor in the pain-compensatory mechanism for fibromyalgia and, accordingly, should be noticeable through TMS assessment variables.

## 2. Materials and Methods

### 2.1. Study Design

This study is a cross-sectional analysis of the baseline data of one ongoing randomized sham-controlled clinical trial, NIH grant R01 AT009491-01A1ClinicalTrials.gov ID: NCT03371225. This trial occurred at the Neuromodulation Center of the Spaulding Rehabilitation Hospital and was reviewed and approved by the Partners Institutional Review Board [15].

### 2.2. Participants

All 108 subjects who completed the baseline visit of the trial until April 2024 were included in the data. Written informed consent was obtained from all enrolled subjects prior to the start of the trial.

The eligibility criteria to participate were: (i) adult subjects between the ages of 18 and 65, (ii) FM pain diagnosis according to the ACR2010 criteria [16], (iii) pain resistance to common analgesics, and (iv) ability to feel pain. The exclusion criteria were any contraindication to tDCS and TMS, taking opiates in high doses, pregnancy, prior neurosurgical procedures, and alcohol or drug abuse within the past six months.

### 2.3. Clinical and Demographic Variables

We used demographic and clinical data collected as the primary and secondary data of the main clinical trial. The choice of variables was determined due to their relevance to fibromyalgia.

#### 2.3.1. Demographic Questionnaire

A standardized form was used to collect age, gender, weight, height, race, ethnicity, side of the pain, hand dominance, duration of fibromyalgia, and educational level. Body Mass Index (BMI) was automatically calculated using the standard formula (BMI = kg/m^2^).

#### 2.3.2. Beck’s Depression Index (BDI)

The 21-question assessment quantifies psychological well-being, depression symptoms, and severity. From these, a score is calculated, with higher values representing more severe depression [17,18].

#### 2.3.3. Patient-Reported Outcomes Measurement Information System (PROMIS)

Subjects were asked to complete the 12-question assessment, and we classified findings according to the sub-sections of pain, fatigue, and anxiety. Higher values indicate increasing severity [19].

#### 2.3.4. Brief Pain Inventory (BPI)

A self-assessment questionnaire that provides information on pain intensity (the sensory dimension) and the degree to which pain interferes with function (the reactive dimension). BPI characterizes subjects’ pain on a scale from 1 (milder) to 10 (more severe). Subjects were asked to complete the whole inventory [20].

### 2.4. Transcranial Magnetic Stimulation Assessment (TMS)

All assessments were performed by a blind assessor with a Bistim2 (Magstim Company LTDA, Carmarthenshire, UK). Using the vertex as a reference, they estimated the spot representing each brain hemisphere’s primary motor cortex (M1). Then, they aimed the coil around the determined spot and attempted to capture the resting motor threshold (MT), defined as the smallest intensity that provokes three motor-evoked potentials (MEPs) with a minimal peak-to-peak amplitude of 100 mV in five tries. The spot where the MT was found was marked with a pen over a swimming cap that subjects wore during the assessment. Afterward, they recorded 10 MEPs, defined as 120% of the MT. The final step was the Paired Pulse assessment, where the investigator applied two pulses: one subthreshold conditioning stimulus (80% MT) followed by one suprathreshold stimulus (120% of the MT) spaced by 2 or 10 millisecond intervals. A total of 30 pulses were recorded, including 10 Paired Pulse recordings with 2 ms, 10 with 10 ms intervals, and 10 single pulses of the MEP. These recordings were used to determine the intracortical facilitation (ICF) and short intracortical inhibition (ICI) for both cerebral hemispheres according to the same formula used in previous studies: ICI = MEP conditioned 2 ms/MEP test, ICF = MEP conditioned 10 ms/MEP test [21].

Additionally, to record the MEPs during the TMS assessment, an Electromyography device was used; electrodes were placed with facilitatory gel over the muscle belly of the first dorsal interosseous, the proximal phalange of the second finger, and at the head of the ulnar bone.

### 2.5. Conditioned Pain Modulation (CPM)

It is a method to evaluate the subject’s capacity to inhibit pain. The test was conducted by a blind investigator and split into two parts: the test stimulus and the conditioned stimulus. First, we determined the pain-60 test, defined as the temperature that induces a pain experience at a magnitude of 60 on a 60–100 Numerical Pain Scale (NPS), using a Peltier thermode (Medoc Advanced Medical Systems, Ramat Yishai, Israel) on the subject’s right forearm and delivering three short heat stimuli (43, 44, and 45 °C) lasting 7 s each. Then, we asked them to rate their pain levels at 10, 20, and 30 s at the pain-60 temperature. The mean of the three wasthen calculated as the test stimulus mean. After a 5 min interval, we performed the conditioned stimulus. The subject’s left hand was immersed in a water bath at 10 to 12 °C for 30 s. Then, the same pain-60 temperature was applied to the right forearm of the subject with the left hand still immersed for 30 s, and the subject rated their levels of pain intensity three more times at the pain-60 temperature at 10, 20, and 30 s. A new mean was calculated for the conditioned stimulus. The CPM value was finally calculated as the difference between the test and conditioned stimulus mean (CPM = preconditioned stimulus − postconditioned stimulus), with higher values representing more pain suppression [22].

### 2.6. Statistical Analysis

The normality of data was assumed based on the central limit theorem [23]. We removed outliers using a two-standard deviation cutoff to reduce the impact of outliers. All missing data were approached using Complete-Case analysis [24]. An initial univariate regression model was built with TMS-collected variables, ICI, ICF, and MT, as the independent variables, while clinical assessments were the dependent variables.

To explore the possible role of BMI as an effect modifier, we have chosen to explore its relationship with the clinical assessments CPM, BPI, PROMIS, and BD and the TMS variables ICI, ICF, and MT (see Table 1). For this, a multiple regression analysis model was used to investigate if BMI was an effect modifier by using an interaction term between these variables; we also adjusted the regressions for age and sex.

If BMI was determined to be an effect modifier, with a *p*-value cutoff of 0.1, we stratified the subjects into two groups according to the predetermined cutoff of 30. Finally, we conducted an analysis with clinical assessments as the dependent variables, TMS variables as the independent variables, and two groups of fibromyalgia subjects, one with BMI above the cutoff and one below. For each group, a linear regression test was performed, and a correlation was determined to be significant when the *p*-value was below 0.05. The statistical analyses and the graph design were performed with R version 4.3.1 [25].

## 3. Results

### 3.1. Participant Demographics

The one hundred and eight subjects included were 87.85% female, 74.07% white, 7.40% black, and 16.67% Hispanic. Their mean age was 47.48 ± 12.09 years, the mean BMI was 28.27 ± 6.3, and the average disease duration was 11.47 ± 8.55 years. Sample characteristics are described in Table 2.

### 3.2. Intracortical Inhibition (ICI)

#### 3.2.1. Beck’s Depression Inventory (BDI)

First, we performed a univariate linear regression between BDI and ICI. A non-significant positive correlation was found (beta-coefficient = 2.99 {95% CI: −1.57; 7.55}, *p*-value = 0.19). See Supplementary Data S1 for more details regarding all of the variables used.

Then, we performed a multivariate linear regression to investigate BMI as an interaction factor. In it, BMI was found to be an effect modifier in ICI’s relationship with BDI (beta-coefficient = −0.88 {95% CI: −1.58; −0.17}, *p*-value = 0.01). See Table 3.

**Figure 1:**
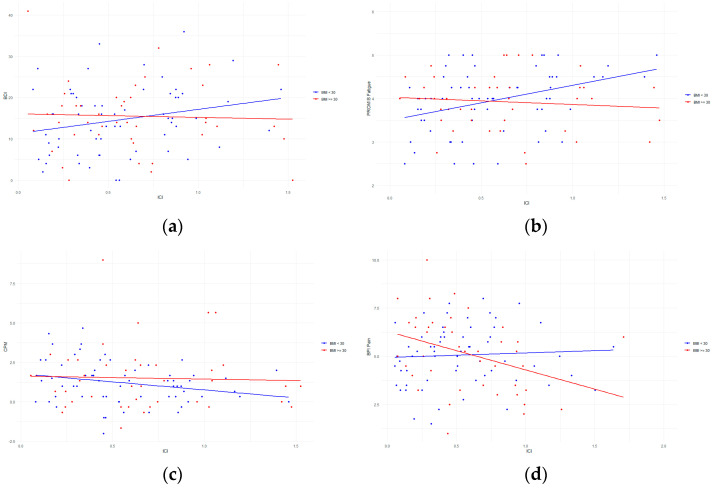
(**a**–**d**) Stratifying BMI for ICI, (**a**) for BDI, (**b**) for PROMIS Fatigue, (**c**) for CPM and (**d**) for BPI pain. On the *X*-axis, we see the neural and clinical variables, while on the *Y*-axis, we see the neurophysiological variables. Two regression lines are represented, with one for the BMI > 30 group in red and one for the BMI < 30 group in blue. Abbreviations: Beck’s Depression Index (BDI), Patient-Reported Outcomes Measurement Information System (PROMIS), Brief Pain Inventory (BPI), conditioned pain modulation (CPM), short intracortical inhibition (ICI), intracortical facilitation (ICF), Body Mass Index (BMI).

After stratification for BMI at a cutoff of 30, we found a correlation in which the group BMI < 30 displays an increase in depressive symptoms, as assessed using the BDI scale, when their inhibitory capacity decreases, as shown by increased ICI amplitudes (beta-coefficient = 6.81 {95% CI: 0.37; 13.24}, *p*-value = 0.04, adjusted R^2^ = 0.074). This finding displays behavior similar to the previous non-significant correlation between BDI and ICI. The group with BMI > 30 does not show this relationship (beta-coefficient = 0.10 {95% CI: −6.66; 6.86}, *p*-value = 0.97, adjusted R^2^ = 0.007). See Figure 1a.

#### 3.2.2. PROMIS Fatigue

There was no statistically significant positive correlation between PROMIS and ICI alone (beta-coefficient = 0.3 {95% CI: −0.06; 0.67}, *p*-value = 0.11). However, BMI was an effect modifier in ICI’s relationship with PROMIS fatigue (beta-coefficient = −0.07 {95% CI: −0.12; −0.01}, *p*-value = 0.01).

When stratifying into two groups, we found another correlation in which the BMI < 30 group has increased fatigue symptoms, as assessed using PROMIS, when their capacity for intracortical inhibition decreases, as shown by increased ICI amplitudes (beta-coefficient = 0.85 {95% CI: 0.32; 1.38}, *p*-value = 0.002, adjusted R^2^ = 0.191). The BMI > 30 group again does not have this relationship (beta-coefficient = −0.17 {95% CI: −0.68; 0.34}, *p*-value = 0.52, adjusted R^2^ = 0.011). See Figure 1b.

#### 3.2.3. CPM

For CPM, we found no statistically significant negative correlation between CPM and ICI (beta-coefficient = −0.64 {95% CI: −1.56; 0.27}, *p*-value = 0.17). On the other hand, we found BMI to be an effect modifier in CPM’s relationship with ICI (beta-coefficient = 0.12 {95% CI: −0.01; 0.27}, *p*-value = 0.07).

By stratifying, we found a correlation in which the BMI < 30 group’s endogenous pain inhibition capacity decreases, as shown by a decreased CPM value; their capacity to inhibit intracortical pulses also reduces, as demonstrated by increased ICI amplitudes (beta-coefficient = −1.23 {95% CI: −2.27; −0.18}, *p*-value = 0.025, adjusted R^2^ = 0.069). The BMI > 30 group again does not have this relationship (beta-coefficient = −0.31 {95% CI: −1.99; 1.36}, *p*-value = 0.71, adjusted R^2^ = 0.058). See Figure 1c.

### 3.3. ICI on the Left Hemisphere

#### BPI

Considering only the ICI of the left hemisphere, we found no statistically significant negative correlation between it and BPI pain (beta-coefficient = 0.02 {95% CI: −0.01; 0.07}, *p*-value = 0.2).

When using BMI as an interaction factor, we found that BMI is an effect modifier for BPI pain (beta-coefficient = −0.14 {95% CI: −0.26; −0.03}, *p*-value = 0.01). This effect maintained itself when controlling for the side most affected by pain. See Table 3.

After stratifying at a BMI cutoff of 30, we found that the group with BMI > 30 showed decreased pain and pain interference, assessed using BPI, as their capacity for intracortical inhibition decreased, as demonstrated by increased ICI amplitudes (beta-coefficient =−1.4 {95% CI: −2.6; −0.15}, *p*-value = 0.03, adjusted R^2^ = 0.172). The BMI < 30 group did not show any statistically significant behavior for either pain or interference (beta-coefficient = 0.53 {95% CI: −0.18; 1.24}, *p*-value = 0.15, adjusted R^2^ = 0.037). See Figure 1d.

### 3.4. Motor Threshold (MT)

#### CPM

In this paper, we found CPM to be negatively correlated with the MT with statistical significance (beta-coefficient = 0.02 {95% CI: −0.00; 0.06}, *p*-value = 0.14), meaning that as the MT increased, the endogenous pain inhibition tended to decrease slightly.

Upon performing a linear regression, we found again that BMI appears to be an effect modifier with its relationship to MT at a BMI cutoff of 30 (beta-coefficient = −0.06 {95% CI: −0.13; 0.00}, *p*-value = 0.058).

**Figure 2:**
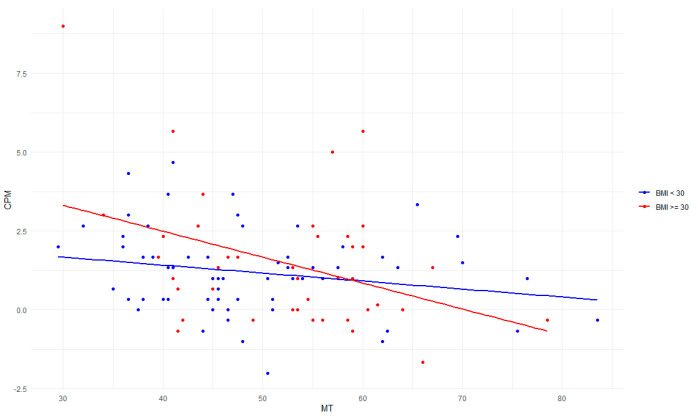
Stratifying BMI for MT. On the *X*-axis, we see CPM, while on the *Y*-axis, we see MT. Two regression lines are represented, with one for the BMI > 30 group in red and one for the BMI < 30 group in blue. Abbreviations: conditioned pain modulation (CPM), Body Mass Index (BMI), motor threshold (MT).

By stratifying, we found in the BMI > 30 group that as their resting motor threshold increases, their endogenous pain inhibition capacity decreases, as shown by a decreased CPM value (beta-coefficient = −0.023 {95% CI: −0.05; 0.00}, *p*-value = 0.131, adjusted R^2^ = 0.069). The BMI < 30 group showed no significant relationship (beta-coefficient = −0.09 {95% CI: −0.16; −0.03}, *p*-value = 0.006, adjusted R^2^ = 0.189). See Figure 2.

## 4. Discussion

### 4.1. Main Findings

Our findings indicate that BMI is an important effect modifier across multiple clinical and neural assessments. In the case of BDI and PROMIS fatigue, as well as CPM, the non-obese group demonstrates a physiologically expected correlation between clinical symptoms and intracortical inhibition (ICI) activity. However, this correlation dissipates within the obese group. This suggests that obesity exerts a disruptive influence on pain mechanisms and their compensatory effects, thereby breaking the physiological balance of compensation and thus worsening pain. Furthermore, our analysis of the resting motor threshold (MT) reveals a trend in the obese group, as higher MT values are associated with weaker endogenous pain control. This observation underscores the potential direct impact of obesity on pain-processing mechanisms.

### 4.2. Intracortical Inhibition (ICI)

Obesity is a chronic inflammatory state that impacts different organs and tissues, including the central nervous system [26]. This process is called neuroinflammation, and it appears to be pivotal in the pathophysiology of metabolic syndrome by affecting the hypothalamus [27]. Still, it also involves other regions of the encephalus, specifically the cortex [28]. At the cellular level, the most significant consequence of this development is apoptosis [29]. Many adipokines and proinflammatory cytokines have been described ascrossing the blood–brain barrier successfully and have even been associated with neurodegeneration [30]. At the structural level, obesity has been shown to impact white and gray matter volume; its effect in regions involved in inhibitory control has also been described [31].

In the context of fibromyalgia, obesity may have an important effect, as this additional chronic inflammation likely supersedes a basal chronic inflammatory state typical of the condition, as shown by previous studies [32,33]. This provides a critical opportunity to assess the effects of inflammation in neural and peripheral markers of pain compensation.

Intracortical inhibition (ICI) is a biomarker in which increased amplitude reveals weaker neuronal inhibition in the cortex. Previous studies have established this measurement as a marker of treatment response in fibromyalgia patients and as a sign of inhibitory maladaptive developments [9]. The rationale behind this correlation is related to GABAergic compensatory activity in the central nervous system and its inhibitory tonus over sensory experiences [34]. Although one of our previous studies could not establish a significant relationship between clinical variables and ICI at baseline [35], it gave us the idea that inside of the complex population of fibromyalgia patients there might be noticeable differences in inhibitory tonus. Among them, being overweight stands out as the most physiologically relevant and prevalent.

We theorized that overweightness and obesity play a disruptive role in ICI compensatory mechanisms. We found evidence for this in BDI, PROMIS fatigue, and CPM, an important marker of endogenous pain control. It appears that individuals within the upper limit of BMI normality display a compensatory relationship to ICI, meaning the more capable their cortex for inhibition, the lower the symptom intensity, which aligns with previous studies [36]. However, for individuals beyond the upper limit of normality, this relationship fades away.

In the case of BDI, it appears that in non-overweight and non-obese individuals, the more intracortical inhibition increases, the fewer depressive symptoms they present. This is in line with the current literature on the subject and could be understood as a compensatory mechanism [37]. The interesting part is that for overweight and obese individuals, this compensatory mechanism ceases to be observable, which so far has not been described in the literature. A similar trend was observed with PROMIS fatigue, with the non-obese group presenting fewer fatigue symptoms as the intracortical inhibition increased, which we also believe to be a compensatory mechanism of a similar kind. Again, BMI over 30 kg/m^2^ proved to be a disruptive factor, and the previous relationship disappeared. Finally, CPM displayed the same behavior in the non-obese group, now in a negative correlation but with a similar interpretation. The more intracortical inhibition increased, the higher the endogenous pain control.

Overall, all of these findings seem to point in the same direction as intracortical inhibition, leading to better clinical results. Accordingly, these findings may play a central role in the use of ICI as a marker for symptom improvement and also unveil a deeper relationship between obesity and fibromyalgia. Given this, we hypothesize that obesity may have an exacerbator effect on fibromyalgia, possibly compromising physiological symptom suppression.

### 4.3. Left Hemisphere Intracortical Inhibition (ICI)

Contrary to the ICI reported above, calculated from the mean of the right and left cortical hemispheres, we also explored unilateral ICI value correlations in the sample. In previous studies, it was shown that, typically, fibromyalgia patients have less capacity for cortical inhibition compared to controls in both hemispheres [38].

Interestingly, in this study, we found a strong correlation between left hemisphere ICI and BPI in the obese population. This is the first time this finding has been reported in the literature. Contrary to the non-obese group, the obese group had an increase in pain symptomatology as the capacity for intracortical inhibition increased, as shown by decreased ICI amplitude values. This means that the idea of ICI representing a compensatory mechanism for pain was inversed in the obese population for the left hemisphere. In the literature, obesity has been described as having a series of structural effects on the brain, affecting different lobes and gyri, many of which are asymmetric, such as, for example, in the left frontal and temporal lobes [39]. However, these asymmetric alterations have not been reported in TMS studies in the past. Therefore, we believe that our findings consist of initial hints at the possibility that obesity may partially compromise pain-compensatory mechanisms, although further investigation is needed.

### 4.4. Motor Threshold (MT)

The resting motor threshold is a measurement of how strong of a pulse has to be applied to evoke a noticeable response. In previous studies, it was found to be increased in subjects with fibromyalgia, meaning that the corticospinal tract required higher amounts of energy to fire compared to healthy controls [38]. We believe that this might be related to the worse motor functionality observed in fibromyalgia patients when compared to controls [40].

In our study, we found a significant correlation between MT and CPM and that obese fibromyalgia subjects show this same finding, as the more hyporeactive the motor cortex, the lesser the pain-suppressor capacity. The overall literature exploring this relationship is still scarce, with two smaller research papers showing no statistically significant findings [41]. Therefore, we believe our paper contributes to this landscape with new valuable information.

Our findings potentially mean that the diminished motor performance thought to relate to MT may be associated with poorer pain suppression, which would make sense from a clinical standpoint, especially in the obese population, where this correlation is starker. Furthermore, this finding gives us the means to quantify the relationship, which could be useful in the area of diagnosing the condition.

### 4.5. Limitations

The main limitation of our study is the inability to establish causality due to its cross-sectional nature. Nevertheless, the findings are still highly relevant to better understand how two extremely prevalent chronic diseases interact, and they can serve as a basis for further investigation. Additionally, it should be noted that TMS assessment is subject to human error, while other instruments are indirect measurements. However, we still believe they provide valuable quantitative information.

## 5. Conclusions

Evidence for obesity being related to greater dysfunction in fibromyalgia patients has been observed in clinical, neurophysiological, and neuroendocrine assessments in the past. However, this study is the first to explore this correlation with the use of a TMS device in a large sample. Our findings above reveal an underlying role for obesity as an effect modifier and disruptor in the neuroplastic processes involved in fibromyalgia syndrome.

## Figures and Tables

**Table 1:** Dependent and independent variables.

Variable Type	1	2	3	4	5	6
Dependent Variables:	BDI	BPI	CPM	PROMIS fatigue	PROMIS pain	PROMIS anxiety
Independent Variables:	MT	ICI	ICF			

The table displays the variables analyzed. Abbreviations: Beck’s Depression Index (BDI), Patient-Reported Outcomes Measurement Information System (PROMIS), Brief Pain Inventory (BPI), conditioned pain modulation (CPM), short intracortical inhibition (ICI), intracortical facilitation (ICF).

**Table 2:** Demographics of the sample.

Characteristic	Values					
Age (mean ± sd)	47.48 ± 12.09					
BMI (mean ± sd)	28.27 ± 6.3					
Duration of disease (mean ± sd)	11.47 ± 8.55					
Sex (%)	87.85% female (n = 94)	12.14% male (n = 12)				
Race (%)	74.07% white (n = 80)	7.40% black (n = 8)	3.70% Asian (n = 4)	9.25% multiracial (n = 10)	4.62% not reported (n = 5)	0.92% Pacific Islander (n = 1)
Ethnicity (%)	77.77% non-Hispanic (n = 84)	16.67% Hispanic (n = 18)	0.05% not reported (n = 6)			
Pain side (%)	50.9% both (n = 55)	24.07% left (n = 26)	25% right (n = 27)			
Education level (%)	1.85% middle school (n = 2)	12.96% high school (n = 14)	79.62% college (n = 86)	5.55% PhD (n = 6)		
Smoking status (%)	29.62% smokers (n = 32)	70.37% non-smokers (n = 76)				
Alcohol status (%)	38.88% drink (n = 42)	61.11% no (n = 66)				

Subject demographics displayed with the characteristics in the left column and their respective distributionsin the right columns. Abbreviations: Doctor of Philosophy (PhD).

**Table 3:** Findings.

Model	Group	Beta-Coefficient	95% CI		R-Squared	*p*-Value
		ICI				
BDI	BMI < 30	6.81	0.38	13.25	0.07	0.04
	BMI > 30	0.10	−6.66	6.87	0.08	0.98
PROMIS Fatigue	BMI < 30	0.85	0.32	1.38	0.19	0.003
	BMI > 30	−0.17	−0.69	0.35	0.01	0.52
CPM	BMI < 30	−1.23	−2.28	−0.18	0.07	0.03
	BMI > 30	−0.32	−2.00	1.36	0.06	0.71
		MT				
CPM	BMI < 30	−0.02	−0.05	0.01	0.07	0.13
	BMI > 30	−0.09	−0.16	−0.03	0.19	0.01
		ICI Left				
BPI Pain	BMI < 30	0.53	−0.19	1.25	0.04	0.15
	BMI > 30	−1.36	−2.57	−0.15	0.17	0.03

Findings for the stratification of patients according to BMI cutoff (kg/m^2^) when the ICI, MT, and ICI left were the independent variables and BDI, PROMIS fatigue, and CPM were the dependent variables. No significant effect was found for ICF or the clinical assessments or for other sections of PROMIS apart from fatigue. Abbreviations: Beck’s Depression Index (BDI), Patient-Reported Outcomes Measurement Information System (PROMIS), Brief Pain Inventory (BPI), conditioned pain modulation (CPM), short intracortical inhibition (ICI), intracortical facilitation (ICF), Body Mass Index (BMI).

## Data Availability

The original contributions presented in the study are included in the article and Supplementary Materials; further inquiries can be directed to the corresponding authors.

## Supplementary Materials

The following supporting information can be downloaded at: https://www.mdpi.com/article/10.3390/jcm13133878/s1, Data S1: Complete analysis results.
